# Separation of fNIRS Signals into Functional and Systemic Components Based on Differences in Hemodynamic Modalities

**DOI:** 10.1371/journal.pone.0050271

**Published:** 2012-11-19

**Authors:** Toru Yamada, Shinji Umeyama, Keiji Matsuda

**Affiliations:** Human Technology Research Institute, National Institute of Advanced Industrial Science and Technology (AIST), Tsukuba, Ibaraki, Japan; University of Cambridge, United Kingdom

## Abstract

In conventional functional near-infrared spectroscopy (fNIRS), systemic physiological fluctuations evoked by a body's motion and psychophysiological changes often contaminate fNIRS signals. We propose a novel method for separating functional and systemic signals based on their hemodynamic differences. Considering their physiological origins, we assumed a negative and positive linear relationship between oxy- and deoxyhemoglobin changes of functional and systemic signals, respectively. Their coefficients are determined by an empirical procedure. The proposed method was compared to conventional and multi-distance NIRS. The results were as follows: (1) Nonfunctional tasks evoked substantial oxyhemoglobin changes, and comparatively smaller deoxyhemoglobin changes, in the same direction by conventional NIRS. The systemic components estimated by the proposed method were similar to the above finding. The estimated functional components were very small. (2) During finger-tapping tasks, laterality in the functional component was more distinctive using our proposed method than that by conventional fNIRS. The systemic component indicated task-evoked changes, regardless of the finger used to perform the task. (3) For all tasks, the functional components were highly coincident with signals estimated by multi-distance NIRS. These results strongly suggest that the functional component obtained by the proposed method originates in the cerebral cortical layer. We believe that the proposed method could improve the reliability of fNIRS measurements without any modification in commercially available instruments.

## Introduction

Continuous-wave functional near-infrared spectroscopy (CW fNIRS) is a simple technique used for cerebral functional imaging, particularly for subjects who are in relatively unrestrained conditions, such as infants without adequate head fixation [1] or ambulatory subjects [2].

In practice, however, the CW fNIRS signal is often contaminated by an optode contact error caused by motion and/or physiological activities other than cerebral function [3]. Although two interrelated factors make up the optode contact error, it can be distinguished relatively easily from other errors. One error is caused by insufficient fixation of optodes to the head, which often generates a rapid drift change or a spiky noise that is accompanied with head motion. Another is caused by an insufficient optode contact to the scalp surface. This leads the detected light flux to be reduced; hence, a large amount of noise can be observed in relevant channels. In contrast, physiological fluctuations caused by cardiac pulsation, respiration, vasomotor action, body motion, posture change, and their interactions produce complicated intrinsic signal changes. Some of them can be evoked by a subject's task execution itself. Such task-evoked physiological fluctuations in a temporal range of several seconds to several tens of seconds were observed to demonstrate parallel changes in oxy- and deoxyhemoglobins in the forehead area with gravitational change [2], in the parietal area with the tilting of the upper body [4], in the occipital area with arm raising [5], in the forehead area during a Valsalva maneuver [2], and in the parietal area during breath holding [4]. Similarly, a prominent change in oxyhemoglobin with a smaller change in deoxyhemoglobin in the opposite direction was also observed in the large parietal area during a motor task [6], in the large area of the forehead during a verbal fluency test [7], and in the parietal area during an anagram task [8]. In all of these cases, there was no, or a very low, functional relation between the measured cerebral region and the kind of task or stimulation being studied. This strongly indicates that various tasks and stimulations evoke hemodynamic responses different from functional hemodynamic responses. Therefore, a subject's motion, posture change, and even the execution of cognitive tasks may cause fluctuations in an fNIRS signal in global cephalic areas.

Cephalic blood circulation involves at least three different mechanisms: the activation of the systemic autonomic nervous system, intracranial autoregulation, and localized neurovascular hemodynamics. Changes in systemic circulation during task execution have been observed, including an increase in the heart rate (HR) and oxygen saturation degradation during a Valsalva maneuver [9], increase in the blood pressure (BP) during arm raising [5], increase in the HR during finger tapping [6], increase in the HR and BP during cognitive and hand-movement tasks [10], increase in the scalp blood flow (SBF) during a verbal fluency test [7], and increase in the HR, BP, and SBF during an anagram task [8]. In addition, changes in intracranial circulation were observed, including an increase in the blood flow velocity in the middle cerebral artery and the degradation of end-tidal 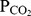 evaluated by PET during cognitive and hand-movement tasks [10]. At present, we do not fully understand how the various kinds of tasks evoke not only the functional hemodynamic response but also the systemic and global cerebral blood circulation.

Conventional techniques of noise reduction, for example, band-pass filter or block averaging, cannot remove such a task-evoked intrinsic contaminant. Thus, if we use CW fNIRS without any knowledge regarding the precise position of the activation area, we need a method to separate the observed signal into the cerebral functional signal and other physiological fluctuations.

Methods to separate a signal into a functional signal and other systemic fluctuations have been proposed; however, both theoretical and practical drawbacks still exist. An adaptive filter [2], [11] and a signal correction method [12], [13] are useful for reducing baseline fluctuations or irregular motion artifacts; however, these may not be very effective for the abovementioned task-evoked systemic fluctuations because these methods are based on the assumption that there is no correlation between a functional signal and systemic fluctuations. Similarly, use of a simple independent component analysis for multichannel fNIRS data [14] is not appropriate for this purpose.

Principal component analysis for multichannel fNIRS data [15] can separate a spatially localized signal from global fluctuations. However, this method requires numerous optodes because of its statistical origin. Furthermore, most of the optodes have to be positioned in regions different from the area of interest, and any optode contact error affects accuracy.

Task-evoked systemic fluctuations can be reduced by a well-designed task [16]. For example, in a finger-tapping task, task-evoked systemic fluctuations are globally observed when the task is designed to include alternate periods of rest and tapping. We can reduce these fluctuations further by using a design that alternates left-hand and right-hand finger tapping without rest. An oxyhemoglobin signal obtained by such a task design shows a temporal change distinctly different from that obtained during a simple task [17]. This may be because the systemic fluctuation is held constant over the two tasks, and in this case, the fluctuation does not correlate with the execution of each task. This type of experiment, equipped with both target and reference tasks, is usually difficult to design and may require a longer execution time. In many cases, it is difficult to verify whether such fluctuations are equivalent between the target and reference tasks.

To overcome the above-mentioned problems, selective detection of the cerebral cortical hemodynamics has been studied with diffuse optical tomography (DOT) [18], CW multidistance (CWMD) NIRS [4], frequency-domain multidistance NIRS [19], and time-resolved (TR) NIRS [20]. These methods can separate the observed signal into the components of the cerebral and other layers. While these methods can successfully detect the functional activity with less contamination, some difficulties exist in implementing them using commercially available fNIRS systems. First, the optode arrangement needs to be modified from its default configuration; however, optode fixtures in most systems are not suited for such a modification. Second, detectors receive light from sources at various distances, and the difference in the detected intensity between near and far detectors is sometimes very large. Therefore, systems need to have a large dynamic detection range to realize accurate measurements. If we employ time-resolved NIRS, the system must be equipped with short-time pulse lasers and time-amplitude converters. Few commercially available systems satisfy these requirements. In this study, we propose a simple new method for separating the fNIRS signal obtained by commercially available systems into a functional signal and other physiological fluctuations.

As mentioned above, we do not have complete information about how the task execution evokes not only the functional hemodynamic response but also the systemic and global cerebral blood circulation changes. Also, even if a fully detailed theoretical model on cephalic blood circulation is given, some of the model's parameters, such as temporal changes in oxygen consumption, the tilt angle of the subject's body, the elasticity of vessels, the density and activity of sympathetic/parasympathetic nerves, or the carbon dioxide concentration may still require measurement by methods other than NIRS. Nonetheless, we can discuss how changes in a different aspect of blood circulation can cause a different hemodynamic result. Here we consider velocity and volume as important aspects of blood circulation. As discussed in later sections, if the velocity and volume of blood flow vary in different kinds of vasculatures (arteries, arterioles, capillaries, venules, and veins), they will cause two distinctively different hemodynamic modalities: cerebral functional hemodynamics in the capillaries of the local cerebral region and a systemic fluctuation in other vessels of the other tissues. These two hemodynamic modalities are characterized by their negative and positive correlation between oxy- and deoxyhemoglobin changes, respectively. Thus, we can use these characteristics to separate the fNIRS signal into functional and systemic components. We simply assume that these correlations are linear, that the observed fNIRS signal is a mixture of the functional and systemic components, and we provide an empirical procedure to determine the coefficients of the linear relationship between the oxy- and deoxyhemoglobin changes of the two modalities. The observed signal was separated into two components based on these coefficients. The proposed method was validated by the simultaneous implementation of both proposed and CWMD methods for functional and nonfunctional tasks. A high congruence between the functional component of the proposed method and the cerebral signal obtained by the CWMD method was observed. We believe that researchers using commercially available systems can successfully separate their fNIRS data into the functional and systemic components and gain the experimental reliability using this simple method.

## Materials and Methods

### Ethics Statement

The study was approved by the Institutional Review Board of the National Institute of Advanced Industrial Science and Technology (Japan). Written informed consent was obtained from the participants.

### Signal separation into functional and systemic components

We can use fNIRS to observe changes in oxy- and deoxyhemoglobin in tissues. In the past, researchers have attempted to ascertain typical shapes of the oxy- and deoxyhemoglobin changes during cerebral functional activation. Early studies reported that the increase in oxyhemoglobin is prominent while the deoxyhemoglobin change is unclear [21], [22]. However, in these studies, task-evoked contaminants were not appropriately accounted for. There are studies that use appropriate experimental designs to reduce task-evoked systemic contaminants, i.e., visual stimulation [23]–[27], tactile stimulation [6], an alternating single-sided motor task [17], the selective detection of the cerebral layer [4], [18], [19], and rodent studies with or without a thinned skull [28]–[33]. These studies reported that stimulation simultaneously evokes a substantial increase in oxyhemoglobin and a considerable decrease in deoxyhemoglobin. This indicates a negative correlation between oxy- and deoxyhemoglobin during cerebral functional activation.

An increase in the regional cerebral blood flow (rCBF) due to neural activation in the corresponding cerebral region was reported more than a hundred years ago [34]. Such an increase in flow causes an overcompensation of blood oxygenation in the capillaries near the activated neurons [35]. The capillaries, which are involved in a major aspect of oxygen delivery, have a diameter smaller than that of a red blood cell. Red blood cells flow through capillaries by deforming their shapes [36]; thus, their flow causes little change in the vessel's volume capacity. Let us assume that the volume capacity of the capillaries is constant, and the oxygen saturation in the capillaries is lower than that in the upstream vessels. If an increase in the rCBF is caused by regional neuronal activation, the blood flow velocity in the capillaries will increase. This will cause the existing blood at a lower oxygen saturation level in the capillaries to be replaced with that at a relatively higher saturation level in the upstream vessels. In this process, oxyhemoglobin will increase and deoxyhemoglobin will simultaneously decrease. Functional activation also involves a change in the cerebral metabolic rate of oxygen (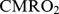). This induces a change in the oxygen delivery, which is mediated by deoxygenation from oxyhemoglobin to deoxyhemoglobin. In this process, the decrease in oxyhemoglobin is always compensated for by an increase in deoxyhemoglobin, even if any temporal change in 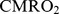 may occur. Consequently, we can expect both hemoglobin changes to occur in opposite directions if they originate from either the rCBF or the 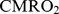 change. This is simply represented by 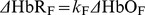 where 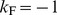. 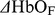 and 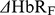 denote the oxy- and deoxyhemoglobin changes in these processes, respectively.

If changes in the regional blood volume (rCBV) occur during a neural-activation-evoked vascular response, they may lead to an increase in the 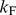 value. Studies using MRI have revealed that neural activation increases not only rCBF by several increments of 10% but the rCBV by several percent [35], [37]–[39]. Tissular origins of the rCBV change are still unconfirmed, but the basic model of the BOLD signal theory attributes the rCBV change to veins [40]. Experiments using MRI showed that the arterial contribution to rCBV is large and the venous contribution is small [41]. A microscopic study showed an arteriole dilation of approximately 10% evoked by electric neuronal stimulation [42]. Such an increase in rCBV will produce only a small increase in the 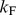 value. In addition, increases in rCBF in the activated area may increase the hematocrit in capillaries because blood flow and hematocrit have a positive correlation in small vessels [43]. This change also produces a small increase in the 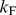 value.

From these findings, we represent the oxy- and deoxyhemoglobin changes evoked by a neural activation as the linear relationship 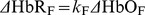 where 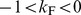. This type of hemodynamic modality is called a modality of cerebral function, and the oxy- and deoxyhemoglobin changes in this modality comprise the functional component.

There is another hemodynamic modality relevant to fNIRS signals. Some researchers have observed that physical activities such as posture change [4], [5], physiological activities such as respiration [2], [4], [44] or gravitational change [2] often cause parallel changes in oxy- and deoxyhemoglobin of the conventional NIRS signal. Posture change or gravitational change may cause tissue hyperemia, including the passive dilation of major vasculatures, such as veins and arteries. A cardiac stroke may be accompanied by a passive compliance of artery and arteriole capacities. Autonomic nerves regulate a vasomotor constriction/dilation in arteries and arterioles, and psychophysiological loads often evoke such a vasomotor response and cause task-evoked systemic fluctuations. Regarding venules, when the blood flow increase due to some physiological reason, the venule will receive more blood from the upstream capillaries and will passively dilate because of vessel compliance. During these processes, arteries, arterioles, venules, and veins change their volume capacities. However, none of them change the blood oxygenation level because they have little oxygen permeability [45]. As a result, oxy- and deoxyhemoglobin levels in these processes will change in parallel, regardless of the physiological origin. This relationship is represented as 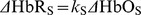 where 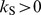. 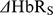 and 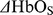 are the oxy- and deoxyhemoglobin changes in these processes, respectively. The coefficient 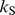 is determined by the blood oxygenation level. For example, 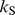 is zero when there is 100% oxygen saturation, 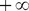 when there is 0% saturation, and a finite positive number in moderate cases. This type of hemodynamic modality is called the modality of systemic fluctuation and the oxy- and deoxyhemoglobin changes in this modality comprise the systemic component.

Based on the above discussion, the CW fNIRS signal (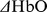 and 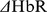) can be represented as a mixture of the functional and systemic components as follows.
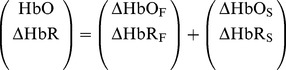
(1)


We suppose a linear relationship in each modality as follows.
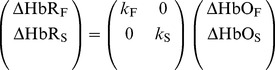
(2)


From the discussion of both modalities, we have 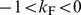 and 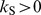. Thus, the following equations are obtained.
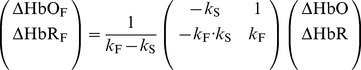
(3)

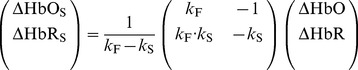
(4)


The coefficients 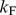 and 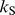 depend on several conditions such as hematocrit, blood oxygen saturation (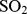), and vascular elasticity. Most of the conditions are under homeostatic regulation, and thus we assume that each coefficient is constant during the usual measuring time and conditions.

### Empirical determination of 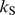 and 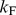


To conduct the signal separation using Eqs. (3) and (4), the coefficients of both hemodynamic modalities, 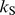 and 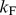, have to be known. In the following section, we propose a procedure to determine them.

The functional component originates mainly from the regional cerebral hemodynamics evoked by neural activation. We surveyed fNIRS studies from the past and utilized studies satisfying two conditions: (1) those that used an appropriate experimental design to exclude physiological signals other than cerebral hemodynamics and (2) those that provided a graph of both oxy- and deoxyhemoglobin changes from the beginning to the end of functional activation. We calculated the value of 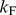 from the graph. A vertically flipped copy of the graph was superposed on the original graph using a drawing software (Illustrator, Adobe Systems), and the original's vertical magnification was manually modulated such that the oxyhemoglobin changes in the original graph visually coincided with the deoxyhemoglobin changes in the copy. The magnification percentage gave 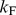. In most cases, the shape of the flipped deoxyhemoglobin graphs was quite similar to that of the original oxyhemoglobin graphs. This indicated that the negative linear correlation between oxy- and deoxyhemoglobin generally held true for the hemodynamics associated with neural activation. The result is shown in Table 1. All values given by the studies fell in a range of 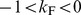. In Table 1, the mean 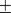 SD of 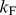 was −0.56
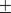
0.12. From the statistical analysis using ANOVA, the 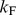 values in Table 1 showed a statistical difference between groups with visual and other kinds of stimulation (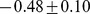 for visual and 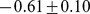 for other kinds of stimulation; 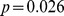). There was no statistical difference in 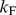 between different subjects and durations. From these analyses, we adopted 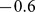 as the universal 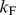 value.

**Table 1:** Estimation of 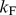 based on existing fNIRS studies.

Reference	Method	Wavelength (nm)	Subject	Stimulation	Duration	*k*_F_
Jasdzewski [23]	CW	682, 830	human	visual pattern reversal	2 s	−0.36
Tang [24]	CW	690, 830	human	visual pattern reversal	4 s	−0.37
McIntosh [19]	FDMD	690, 830	human	visual pattern reversal	30 s	−0.40
Siegel [33]	DOT	690, 830	rat	forepaw stimulation	6 s, 30 s	−0.48, −0.48
Villringer [25]	CW	775, 825, 850, 904	human	flash light exposure	50 s	−0.51
Lindauer [28]	CW	500–590	rat	whisker deflection	4 s	−0.53
Obrig [26]	CW	725–940	human	visual pattern reversal	30 s	−0.56
Zeff [18]	DOT	750, 850	human	visual pattern reversal	10 s	−0.56
Boden [17]	CW	760, 850	human	alternating finger tapping	20 s	−0.57
Dunn [29]	OIS	560–610	rat^a^	forepaw stimulation	10 s	−0.57
Colier [27]	CW	775, 848, 901	human	visual pattern reversal	10 s	−0.61
Berwick [30]	OIS	505–602	rat^a^	whisker stimulation	1 s	−0.63
Yamada [4]	CWMD	776, 809, 850	human	finger tapping	20 s	−0.66
Huppert [31]	CW	560, 570, ..., 610	rat^a^	whisker stimulation	20 ms	−0.66
Franceschini [6]	CW	690, 830	human	finger tactile	20 s	−0.67
Sheth [32]	CW	570, 610	rat^a^	hind-paw stimulation	2 s	−0.82

CW, continuous-wave NIRS; FDMD, frequency-domain multidistance NIRS; DOT, defuse optical tomography; OIS, optical-imaging system; CWMD, continuos-wave multidistance NIRS; 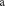, thinned skull.

The coefficient 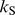 of the systemic component has the relationship 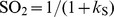 with 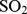. It is known that oxygen saturation levels differ among vessels. For example, under normal conditions, the saturation level in arteries is greater than 95%, while that in veins is approximately 70% [46], and it decreases to approximately 50% after intense physical activity. These saturation levels correspond to 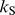 of less than 0.053, 0.43, and 1.0, respectively.

The coefficient 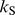 may vary according to the type of task. If a task containing a psychophysiological load is executed, it may change the arterial blood pressure, respiration rate, and vasomotor action. These changes cause blood volume changes mainly in the arteries and arterioles. Since blood in the arteries and arterioles has a high oxygen saturation level, 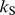 should be small. In contrast, hyperemia induced by a posture change gives a larger 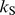 value because a passive volume capacity change can occur not only in arteries but also in veins having lower oxygen saturation levels. Furthermore, different 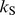 values are expected if the intensity of physical activity is varied. Thus, unlike 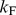, we cannot expect a universal value of 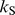 under various task conditions.

In our model, we assume that capillaries mainly generate the functional component and arteries and veins generate the systemic component. Since these components originate from different vessels and different hemodynamic modalities, we assumed a high statistical independence between them. Thus, we determined 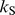 by minimizing the mutual information between these components. The mutual information 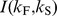 is given as follows.
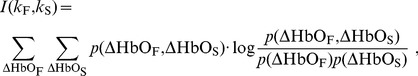
(5)where 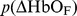 and 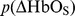 represent the probability density functions of 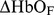 and 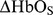, respectively, and 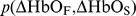 represents a joint probability density function of 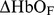 and 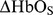. These probabilities are estimated by the normalized histograms of 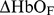 and 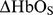, and the normalized joint histograms of 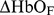 and 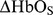. Histograms are calculated from Eqs. (3) and (4) when 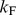 and 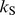 are given. Here we fix 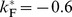 based on the discussion on Table 1. Thus, if we set 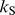, the mutual information 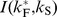 can be calculated by Eq. (5). By enumerating 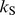 in 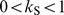 we determine 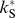, which minimizes the mutual information.

### fNIRS data acquisition

**Figure 1:**
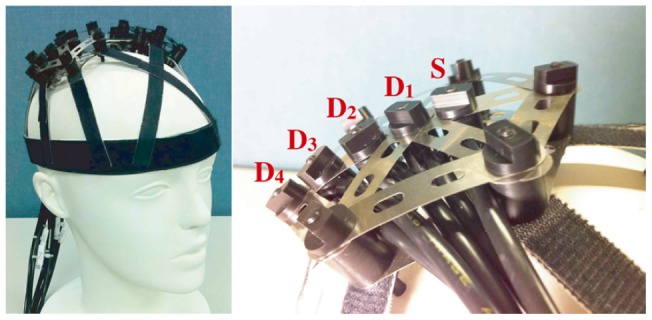
Specially designed optodes and their holder system. Optode arrays consisting of a source (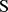) and four detectors (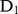
–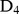) were fixed directly above the left and right primary motor areas. The detector optodes on both sides were linearly aligned at distances of 10, 20, 30, and 40 mm from the source optode.

We used an OMM-3000 system (Shimadzu Corp., Japan) with our specially designed optodes and holder system [47] for the CW fNIRS measurements. As shown in Figure 1, the optode arrays consisting of a source and four detectors were fixed directly above the left and right primary motor areas. The detector optodes on both sides were linearly aligned at distances of 10, 20, 30, and 40 mm from the source optode. Optical attenuation recorded at wavelengths of 780, 805 and 830 nm and a frequency of 40 Hz were down-sampled at 10 Hz and filtered using a low-pass filter of 1.0 Hz.

Oxy- and deoxyhemoglobin changes in the CW fNIRS signals were calculated using a pseudo-inverse of the molar absorption coefficient matrix. The molar absorption coefficients were taken from the literature [48]. Oxy- and deoxyhemoglobin changes in the proposed modalities were calculated by Eqs. (3) and (4). The CWMD method [4] was used to compare the hemodynamics in the functional component using the proposed method with those in the cerebral gray matter layer. The data obtained by the optodes at distances of 20 and 30 mm from the source optode were used for the CWMD method.

### Experimental procedures

The study included seven healthy adult volunteers. The primary motor area of four participants (P1, P4, P5 and P7) was initially identified by fMRI during a finger-tapping task (detailed below). The T1-weighted images and echo planar images were measured using MR equipment (MR Signa 3.0T, GE Yokogawa Medical Systems KK). The T-contrast images of left- and right-hand-finger tapping against rest periods were obtained using SPM5 (see http://www.fil.iion.ucl.ac.uk/spm/). The activation area and the Cz position of an EEG 10–20 system were identified by overlying the T1-weighted image. Each optode array was located directly above the activation area relative to the Cz position.

Participants P2, P3 and P6 were exempted from fMRI because of a request to stop (P2 and P6) and because of a metal implant (P3). These three participants participated with optodes set around positions C3 and C4 in the parietal area. Participant 3 participated only in the nonfunctional tasks. In total, the nonfunctional tasks were performed by seven participants and the functional task by six. Participants were seated in a chair and were instructed to perform the tasks described below.

Two types of nonfunctional tasks were performed: voluntary tilting of the upper body forward by approximately 30 degrees and holding one's breath. In each task, the block conditions were alternated through a task period (20 s) and a rest period (20 s) using visual and auditory cues. A complete session consisted of an initial reference rest period (20 s) followed by five task/rest sequences.

In the functional task, the participants were instructed to tap their thumb with the index finger at a frequency of 4 Hz. The block conditions were alternated using visual and auditory cues in the following order: a left-hand finger-stimulation period (20 s), a rest period (20 s), a right-hand finger-stimulation period (20 s), and a rest period (20 s). A complete session consisted of an initial reference rest period (20 s) and five repetitions of the block sequence (left finger tapping, rest, right finger tapping, and rest) without any interruption.

### Data Analysis

To evaluate the performance of the proposed method for detecting the cerebral functional activity, we conducted a paired t-test for the difference in oxyhemoglobin change between left and right finger tapping by the following procedure. We averaged oxyhemoglobin changes during tasks and their preceding rest periods (each was of 10 s duration), where we adopted a 10 s temporal offset in each period to take into account the transient phase of the functional hemodynamics. The difference between the task and rest was averaged for five trials of each tapping side. For the results, the difference between left- and right-tapping was examined by the paired t-test.

To examine the agreement of the temporal shapes between the functional component of the proposed method and the hemodynamics obtained by the CWMD method, a Pearson product-moment correlation coefficient was calculated from the data of the finger-tapping task.

To analyze the periodical characteristics of hemodynamic changes, we conducted Fast Fourier Transformation (FFT) on the data that had not been passed by a low-pass filter of 1.0 Hz.

## Results and Discussion

### Stability of 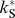 estimation

**Figure 2:**
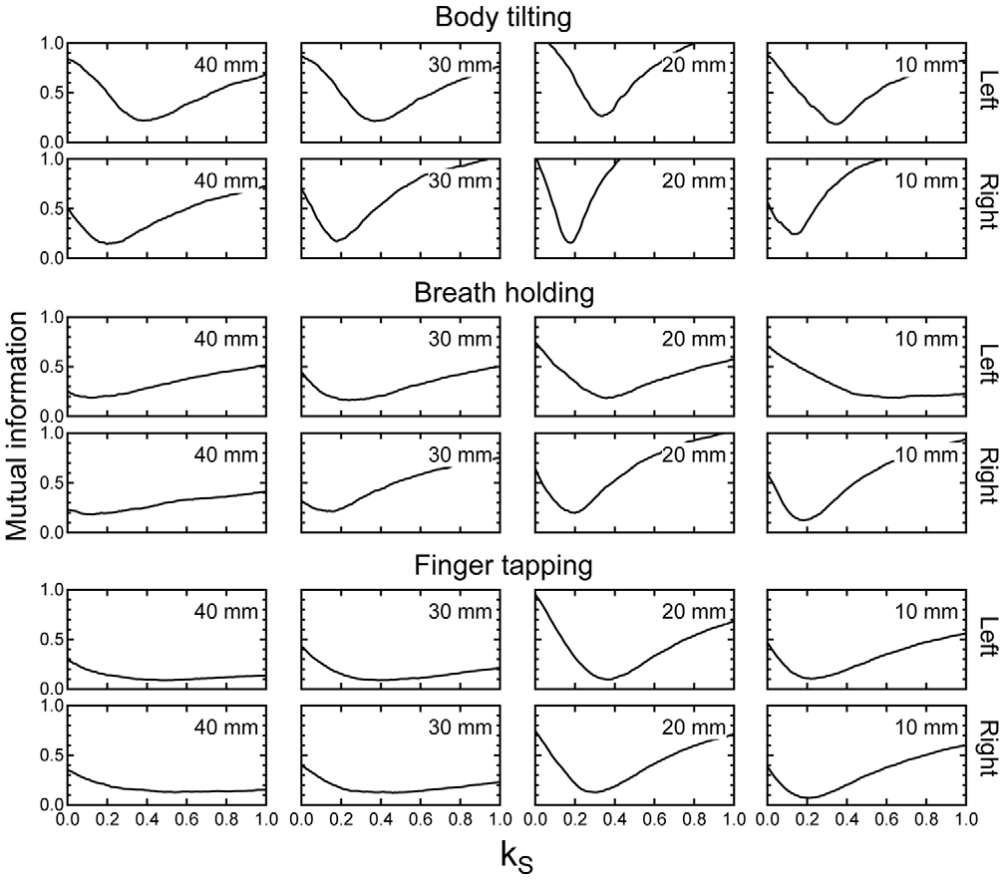
The 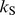
 **dependency of the mutual information** 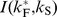 **for participant 1.** Upper two rows: body-tilting task. Middle two rows: breath-holding task. Bottom two rows: finger-tapping task. Left and Right indicate the measurement positions. 10 mm, 20 mm, 30 mm, and 40 mm indicate the source-detector distances.

Figure 2 shows the 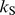 dependency of the mutual information 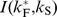 for participant 1 at various source-detector distances and tasks. Most cases in Figure 2 show a unique minimum point for mutual information, which means that the minimization of mutual information provides a stable estimation of 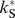. The statistical analysis of the 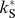 value for each participant using repeated-measures ANOVA showed the following significant differences: left/right positions of participant 1 (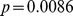) and 6 (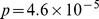), the kind of task being undertaken by participants 4 (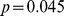) and 6 (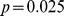). These tendencies may not be universal because no statistical significance was observed in the overall experimental analysis using two-factor factorial ANOVA. Rather, these may be influenced by differences in vessel arrangements in the observed areas. Changes in vessel capacity could vary depending on the type of task. For example, body tilting induces hyperemia; thus, it may lead to more changes in veins than in arteries. If the populations of arteries and veins under observation are different, it will result in a different 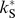 value.

**Figure 3:**
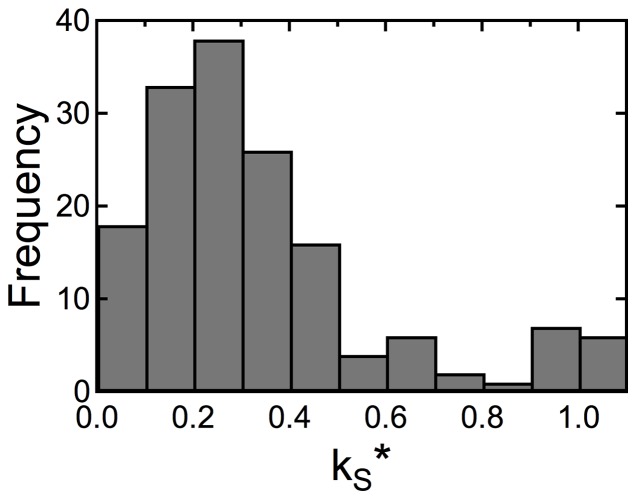
Histogram of estimated 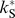
 **values from all experiments.**

A histogram of the estimated 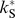 values from all experiments is shown in Figure 3. This figure shows that most of the estimated values are in the range of 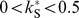. Since the tasks used in this study cause only mild physical and physiological loads on the participants, the oxygen saturation level of veins would be approximately 70% during the tasks. Therefore, if all of the systemic fluctuations originate from veins, 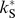 should be approximately 0.4, based on the previous discussions. However, if the systemic fluctuations originate from arteries, then 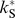 should be almost 0. The actual measurement should be in the middle. The actual histogram agrees with this prediction. In addition, an accumulation at 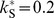 in the histogram suggests that arteries and veins contribute almost equally to the systemic component. A small peak around 1.0 was observed. To determine 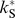, we calculated the mutual information in ascending order of 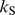 in the rage 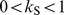. Where the true value of 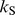 is larger than this range, the 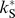 will be estimated as 1. The peak at 1.0 reflects this. However, the population of this peak is very minor and may not overturn the above assumption. Based on these results, we believe that 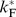 and 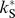 could be accurately estimated by the proposed method.

### Hemodynamic changes during task executions

**Figure 4:**
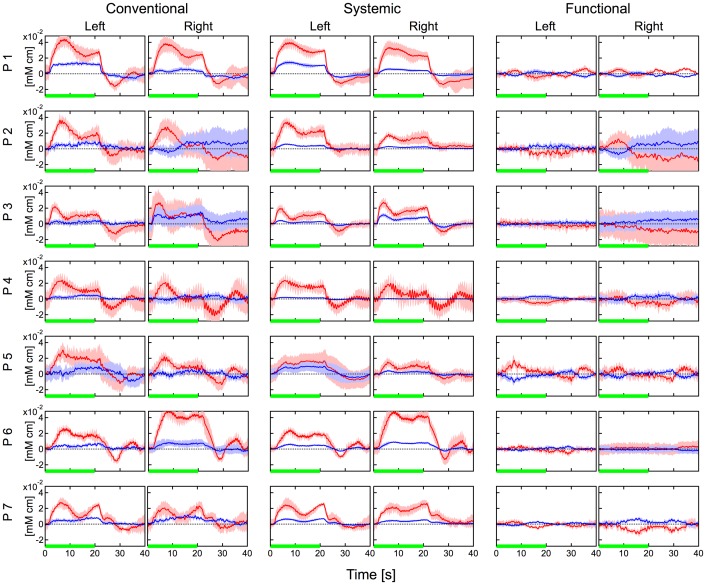
Hemodynamic changes during the body-tilting task for all participants. Left two columns: hemodynamics estimated by the conventional method. Middle two columns: systemic component by the proposed method. Right two columns: functional component by the proposed method. Data were block averaged. Distance between source and detector was 30 mm. Red and blue lines indicate oxy- and deoxyhemoglobin changes, respectively. Red and blue bands indicate SDs for oxy- and deoxyhemoglobin changes, respectively. Green line indicates the task period. “Left” and “Right” indicate the measurement positions.

**Figure 5:**
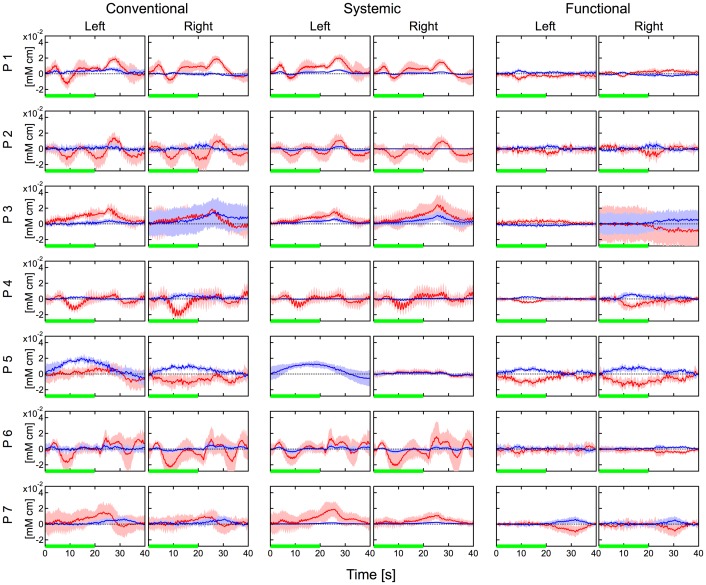
Hemodynamic changes during the breath-holding task for all participants. Left two columns: hemodynamics estimated by the conventional method. Middle two columns: systemic component by the proposed method. Right two columns: functional component by the proposed method. Data were block averaged. Distance between source and detector was 30 mm. Red and blue lines indicate oxy- and deoxyhemoglobin changes, respectively. Red and blue bands indicate SDs for oxy- and deoxyhemoglobin changes, respectively. Green line indicates the task period. “Left” and “Right” indicate the measurement positions.

**Figure 6:**
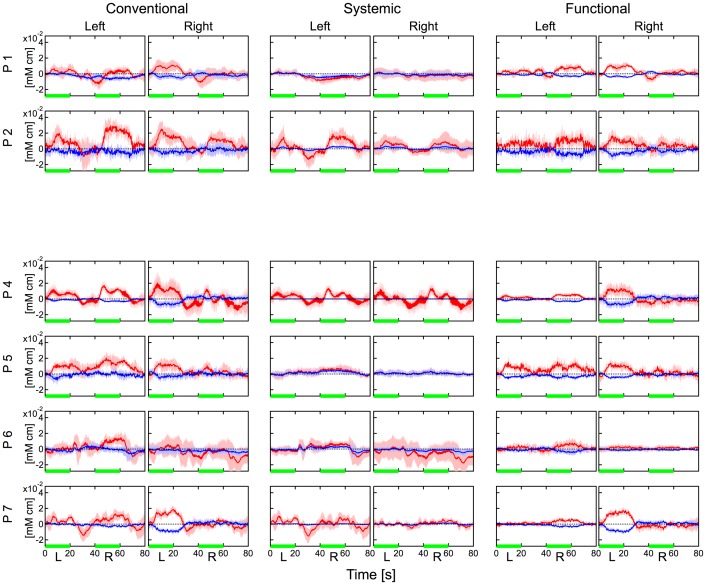
Hemodynamic changes during the finger-tapping task for participants except participant 3. Left two columns: hemodynamics estimated by the conventional method. Middle two columns: systemic component by the proposed method. Right two columns: functional component by the proposed method. Data were block averaged. Distance between source and detector was 30 mm. Red and blue lines indicate oxy- and deoxyhemoglobin changes, respectively. Red and blue bands indicate SDs for oxy- and deoxyhemoglobin changes, respectively. Green line indicates the task period. “Left” and “Right” indicate the measurement positions. “L” (left) and “R” (right) indicate the side used during finger tapping.

Figures 4, 5 and 6 show hemodynamic changes estimated by the conventional method (left two columns) and hemodynamic changes in the systemic components (middle two columns) and functional components (right two columns) estimated by the proposed method during the body-tilting, breath-holding, and finger-tapping tasks for all participant, respectively. The source-detector distance of 30 mm was used. As reported previously [4], in the conventional method large oxyhemoglobin changes were observed in the left and right positions and for almost all participants during all kinds of task execution. In case of the body-tilting task, deoxyhemoglobin changes during the task were often comparatively smaller but parallel with oxyhemoglobin changes. However, in case of the breath-holding task, the hemodynamics varied for each participant and the parallel changes were not very clear. In many cases of the body-tilting and breath-holding tasks, the systemic component in the proposed method shows hemodynamic changes similar to those observed in the conventional method. In contrast, the changes of the functional component are relatively small. This indicates that the functional component is minimally influenced by the non-functional tasks. In the finger-tapping task, the hemodynamics recorded by the conventional method often did not show a clear signal laterality corresponding to the side of the finger tapping because of unstable baselines. The hemodynamic changes of the functional component in the proposed method showed a simultaneous increase in oxyhemoglobin and a decrease in deoxyhemoglobin on the contralateral side of the finger tapping in many cases. These tendencies were examined with a paired t-test and are summarized in Table 2. In many cases in Table 2, the signal laterality was more significant in the functional component than that in the conventional data.

**Table 2:** Paired t-test for the difference in oxyhemoglobin change between left and right finger-tappings.

	Conventional	Conventional	Systemic	Systemic	Functional	Functional
Participant	Left	Right	Left	Right	Left	Right
P1	−0.6465	1.343	0.97580	−0.2331	−2.029	2.235*
P2	−3.673*	0.7312	−2.893*	−0.8236	−1.842	4.148*
P4	−2.093	1.472	−1.171	−1.091	−3.302*	5.109*
P5	−0.9369	4.828*	−0.5803	−0.3060	−1.115	4.202*
P6	−1.089	1.074	−0.1817	0.8092	−3.131*	2.226*
P7	−0.9161	3.968*	0.03681	−0.5551	−2.223*	4.589*

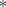
, significant level higher than p = 0.05. Here, the correction for multiple comparisons was not considered. A detailed discussion is presented in the results and discussion section.

**Figure 7:**
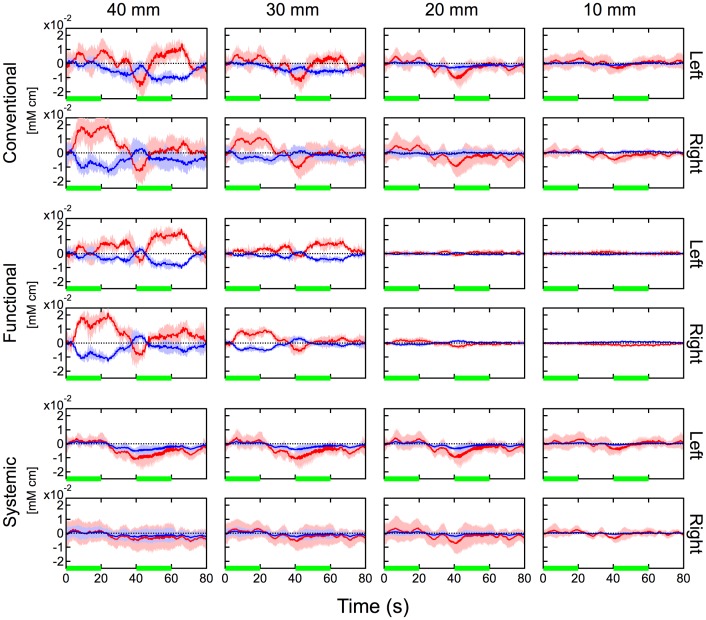
Hemodynamic changes at various source-detector distances during the finger-tapping task for participant 1. Upper two rows: hemodynamics estimated by the conventional method. Middle two rows: functional component by the proposed method. Bottom two rows: systemic component by the proposed method. Data were block averaged. Red and blue lines indicate oxy- and deoxyhemoglobin changes, respectively. Red and blue bands indicate SDs for oxy- and deoxyhemoglobin changes, respectively. Green line indicates the task period. “L” (left) and “R” (right) indicate the side used during finger tapping. “Left” and “Right” indicate the measurement positions. “10 mm,” “20 mm,” “30 mm,” and “40 mm” indicate the source-detector distances.

The dependency of signal amplitude against the source-detector distance in the hemodynamic changes during the finger-tapping task by participant 1 is shown in Figure 7. A simultaneous increase in oxyhemoglobin and a decrease in deoxyhemoglobin on the contralateral side of the finger tapping was observed when the source-detector distance was 30 or 40 mm. Such signals were rarely observed when the distance was 10 or 20 mm. However, the hemodynamic changes of the systemic component showed baseline drifts and task-evoked increases in both oxy- and deoxyhemoglobin, regardless of the finger used to perform the task and even in cases where the source-detector distances were 10 and 20 mm. This difference in dependency on the source-detector distance between two components will be discussed in a later part of this section.

### Comparison of hemodynamics obtained by the conventional, proposed, and CWMD methods

**Figure 8:**
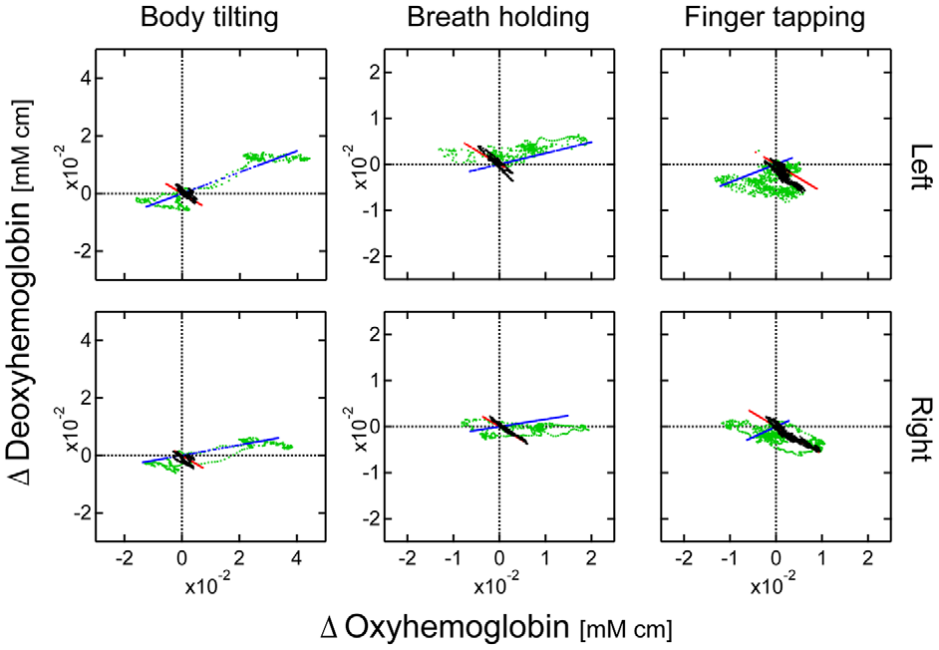
Hemodynamic trajectories of the three methods for participant 1. Data during the three kinds of tasks are shown. Green line: the conventional method. Red and blue lines: the functional and the systemic components of the proposed method. Black line: the CWMD method. “Left” and “Right” indicate the measurement positions.

The CWMD method can estimate hemodynamics in the cerebral gray matter layer. In a previous study [4], we showed that the distance of 20 mm as a reference and of 30 mm as a brain detection are most effective in canceling both the changes in absorption and scattering in the superficial layers. Therefore, we applied this method to the data obtained at source-detector distances of 20 and 30 mm. In Figure 8, different hemodynamic trajectories obtained by CW fNIRS, the proposed, and the CWMD methods are shown for all three tasks of participant 1. The oxy- and deoxyhemoglobin changes are represented as the x- and y-coordinates, respectively. The conventional and proposed methods were applied to the data at a source-detector distance of 30 mm.

**Figure 9:**
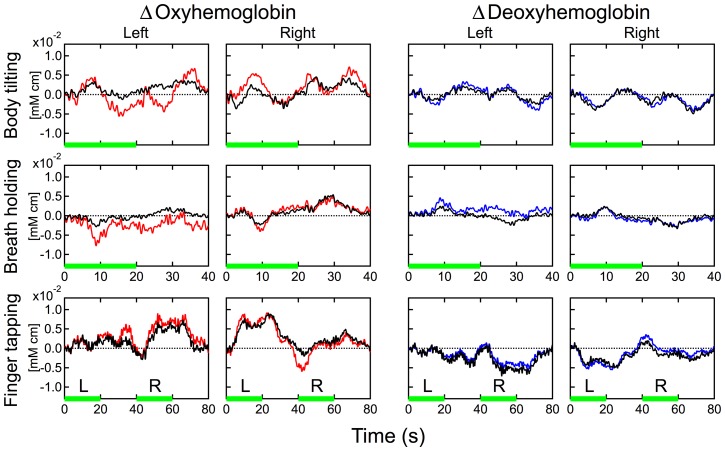
Comparison of estimated temporal hemodynamics of the functional component and the CWMD result. Data during the three kinds of tasks for participant 1 were used. Red and blue lines correspond to oxy- and deoxyhemoglobin changes of the functional component, respectively. Black line: the corresponding hemoglobin changes measured by the CWMD method. The CWMD hemodynamics was rescaled to obtain the best fit to that of the functional component under each condition. “Left” and “Right” indicate the measurement positions. “L” (left) and “R” (right) indicate the side used during finger tapping.

The trajectories of the CWMD method consistently indicate a negative linear relationship between oxy- and deoxyhemoglobin changes, whereas those of the conventional method varied according to the task conditions and were different from those of the CWMD method. The trajectories of the functional component originated from the assumed linear relationship with a coefficient of 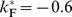. This slope closely matched the trajectories of the CWMD method in all cases. In our measurements, we did not explicitly give the path length. Thus, with both methods, changes in the different hemoglobin levels were given in the same units: 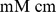. However, the path length with the CWMD method was related to a partial path length in the gray matter layer, while those with the conventional and the proposed methods based on the modified Beer-Lambert law were total path lengths. Therefore, values derived by the CWMD and proposed methods were scaled differently and could not be directly compared with each other. To examine the temporal similarity of these hemodynamics, we rescaled the hemodynamics of the CWMD method to obtain the best fit to the hemodynamics of the functional component. The results are shown in Figure 9. Temporal changes in oxy- and deoxyhemoglobin obtained by the two different methods highly coincided with each other. A Pearson product-moment correlation coefficient between oxy-/deoxyhemoglobin in these two hemodynamics during finger-tapping task for each participant was calculated and summarized in Table 3. Most of values in Table 3 indicated a high correlation between the two hemodynamics. These results strongly suggest that the functional component originates from the cerebral gray matter layer.

**Table 3:** Correlation coefficients between the functional component by the proposed method and the resultant by the CWMD method.

	Left	Left	Right	Right
Participant	OxyHb	DeoxyHb	OxyHb	DeoxyHb
P1	0.932	0.976	0.951	0.937
P2	0.890	0.958	0.754	0.942
P4	0.805	0.948	0.917	0.933
P5	0.280	0.601	0.914	0.913
P6	0.445	0.479	0.490	0.817
P7	0.882	0.811	0.853	0.860

### FFT analysis

**Figure 10:**
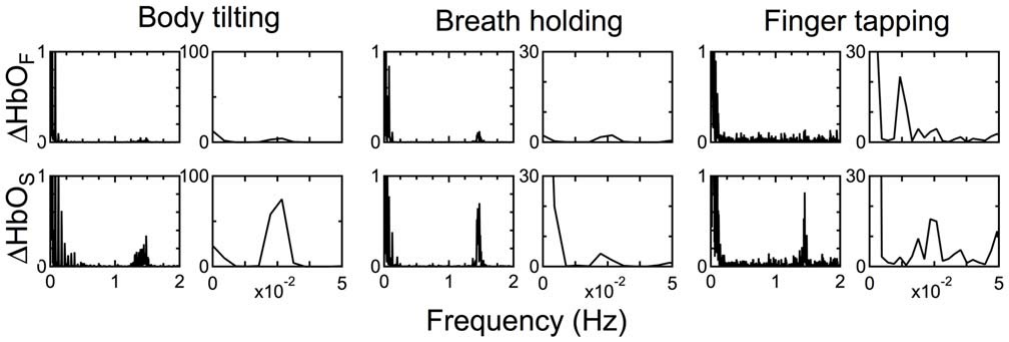
FFT analysis of the functional and systemic components of the three tasks for participant 1. For detailed analysis, the low-frequency range is also shown in the second, fourth, and sixth columns.

We introduced two hemodynamic modalities in this study. If each modality originates from a different physiological activity, we may be able to find a frequency component in the spectrum that is specific to each activity. Figure 10 shows the FFT analyses of the oxyhemoglobin changes in the functional and systemic components for the three tasks for participant 1. In this case, we did not apply a low-pass filter of 1.0 Hz to the original optical attenuation data. All spectra of the systemic component showed obvious peaks at approximately 1.5 and 0.025 Hz. The peak at 1.5 Hz may be caused by a change in the vessel volume capacity from cardiac strokes. The peak at 0.025 Hz coincided with the frequency of task repetition of body tilting, breath holding, and finger tapping regardless of which finger is being tapped. This indicates that hyperemia evoked by posture change and vasomotor responses caused by physical and/or psychophysiological loads lead to changes in the vessel volume capacity. These peaks were barely detected in the spectra of the functional component. Among them, the spectrum of the finger-tapping task has a distinctly larger peak at the half frequency of 0.025 Hz (0.0125 Hz) and a relatively smaller peak at 0.025 Hz. The frequency of the larger peak coincided with the repetition frequency of the left-right alternation of tasks. These characteristics were also found in for the data for other participants. This indicates that the functional component separated by the proposed method successfully represents the cerebral functional hemodynamics. This is also supported by the result shown in Figure 9, in which the functional component closely coincided with the CWMD result.

### Difference in source-detector distance dependencies of optical attenuation between the two modalities

In our model of the two hemodynamic modalities, we did not introduce any assumption about the spatial origin of each modality. However, Figures 6 and 7 clearly show the difference in spatial localization between the two components. Regarding the laterality, the systemic components of all tasks were always observed at both sides of detection, while the functional components were observed mainly at the contralateral side of the tapping finger. In Figure 7, regarding the dependency on the source-detector distance, hemodynamic changes in the systemic component had similar shapes, even when the source-detector distance was different. In contrast, hemodynamic changes of the functional component at 10 mm and 20 mm were barely detected while those at 30 and 40 mm were observed and their shapes were similar to each other. In previous studies using Monte Carlo simulation [4], we investigated dependencies of optical partial path lengths in the cerebral gray matter layer, that is, the sensitivity for detecting cerebral function against the source-detector distance. Simulations indicate that the detection using less than 20 mm of the source-detector distance have little sensitivity for detecting hemodynamics in the cerebral gray matter layer. The tendency in signal amplitude against the source-detector distance in Figure 7 indicates good agreement with this simulation result. Therefore, we consider that hemodynamic changes in each component have similar shapes but the dependency of amplitude on distance differs between the two components. Consequently these results indicate that each hemodynamic modality originates from a tissue layer clearly different in depth and bilateral uniformity.

**Figure 11:**
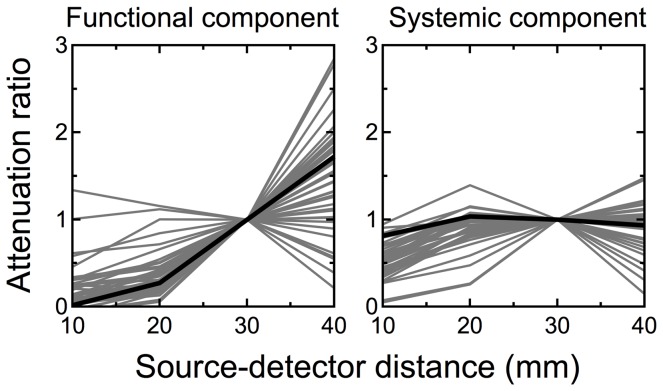
Optical attenuation ratio of the functional and systemic components across different source-detector distances. Optical attenuation changes of the functional and systemic components at a wavelength of 780 nm were calculated by using an absorption coefficient matrix [48]. Ratios in the optical attenuation changes against that at the distance of 30 mm was calculated by a least mean square method such that the shape of optical attenuation change at the distance best coincided with that at 30 mm when that was multiplied by the ratio. A gray line shows ratios across different source-detector distances for each task and each participant. Lines for all tasks for all participants are presented. Black lines indicate the simulated partial optical path lengths of the gray matter (left figure) and scalp (right figure). All values were normalized by the value at the source-detector distance of 30 mm.

Figure 11 shows the ratio of optical attenuation changes in the two modalities over four source-detector distances. Gray lines in each modality indicate the ratio of attenuation changes at a wavelength of 780 nm for all tasks and all participants. Values are normalized at 30 mm. An optical attenuation change is the product of the optical path length and hemodynamic change. If we assume an optical multilayer model where the hemodynamic change is uniform in each optical layer, the optical attenuation is proportional to the optical path length. Therefore, the figure gives the source-detector distance dependency of the optical path length for each modality, and the dependencies of the two components showed a clear difference from one another.

The same figure shows black lines as the source-detector distance dependency of the directly simulated partial optical path length in the scalp and the cerebral gray matter layers. These values were calculated at a wavelength of 780 nm by a Monte Carlo simulation. We used a public code [49] and the optical properties of tissues from the literature [50]. The result is consistent with an existing simulation study [51]. The dependencies of the partial optical path length in the scalp and cerebral gray matter layers are similar to those of the optical attenuation of the systemic and functional components, respectively. This strongly suggests that the functional component originates from the cerebral gray matter layer and the systemic component originates mainly from the scalp layer.

### Stability of the signal separation against 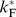 deviation

**Figure 12:**
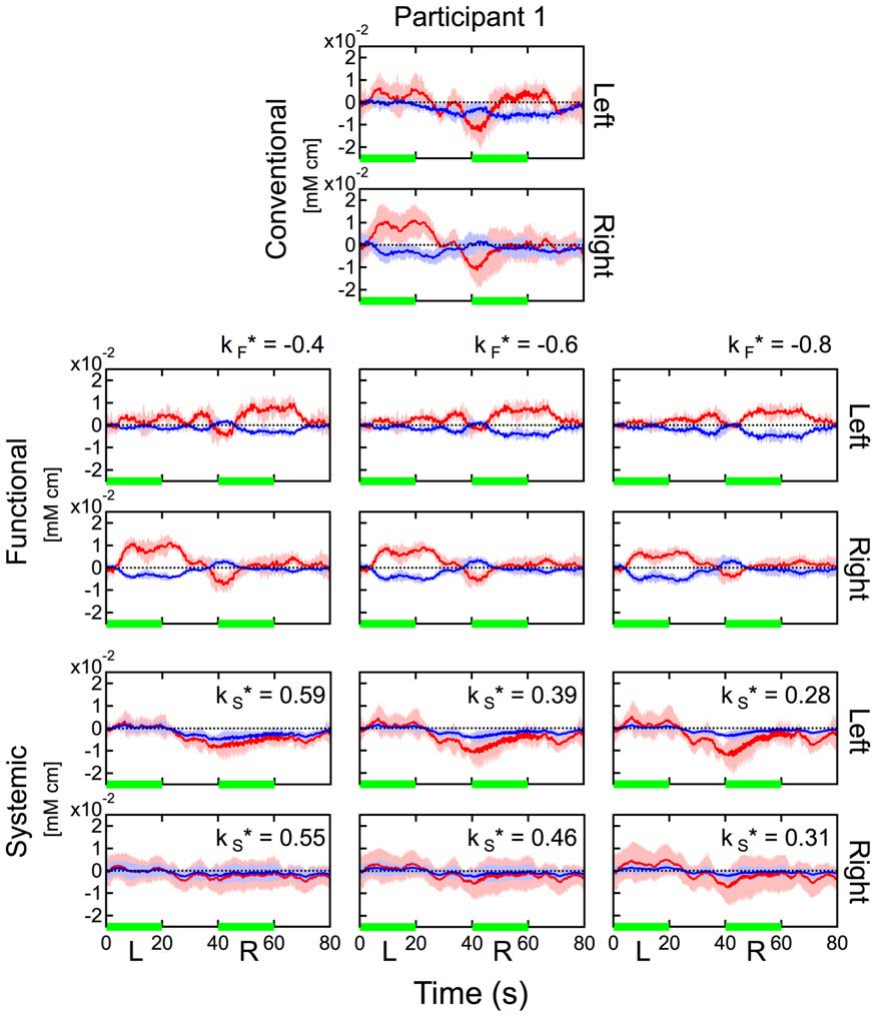
Difference in signal separation under three different 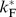
 **conditions.** Values of 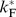: −0.4 (left column), −0.6 (middle column), and −0.8 (right column) were used for the data at a source-detector distance of 30 mm during the finger-tapping task for participant 1. Upper two rows: hemodynamics estimated by the conventional method. Middle two rows: functional component by the proposed method. Bottom two rows: systemic component by the proposed method. Data were block averaged. Red and blue lines indicate oxy- and deoxyhemoglobin changes, respectively. Red and blue bands indicate SDs for oxy- and deoxyhemoglobin changes, respectively. Green line indicates the task period. “Left” and “Right” indicate the measurement positions. “L” (left) and “R” (right) indicate the side used during finger tapping.

In this study, we fixed 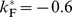 based on the studies listed in Table 1. However, it is unknown whether this value is adequate both theoretically and empirically. For instance, some studies listed in Table 1 used wavelengths that were different from those in our study, and most of these studies did not consider the wavelength dependency on the optical path length. Therefore, crosstalk errors may be included in the list. We estimated the magnitude of the crosstalk error to be approximately 10% in a previous study [52]. In addition, 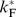 could differ with species, kinds of stimulus, and stimulus duration. From the statistical analysis using ANOVA, the 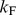 values in Table 1 showed a significant difference between groups with visual and other kinds of stimulation. If 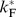 deviated from its true value for these reasons, the estimated 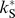 value could also have deviated, and the separated signal components could have been deformed. Therefore, we examined how the different values of 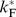 affected the estimations of 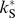 and the results of signal separations. We used the case of the finger-tapping task for participant 1 with a source-detector distance of 30 mm. These results are shown in Figure 12. By varying the 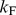 values, the estimates for 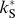 values changed, but the temporal shapes of both functional and systemic components separated by our method varied only marginally. In particular, we could clearly observe the signal laterality in the functional component when any 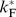 value in the range of 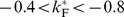 was used. The same data analysis was conducted for the other participants. Similar tendencies with Figure 12 were observed there. The range in 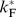 used for this analysis was much larger than the range of errors caused by crosstalk or stimulation type. Therefore, we consider that the estimation error for 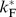 values did not seriously affect the separation of the functional component in cases of healthy subjects.

### Other issues and future works

As shown above, we examined the stability of the proposed method with regard to the stability of 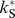 estimation and the robustness against 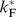 deviation. These results indicate that the proposed method can separate fNIRS signals into systemic and functional components with high stability. However, the temporal stability of the hemodynamic modalities, i.e., the temporal stability of 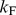 and 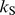, is also important in applying the proposed method. Physiological parameters such as baseline hematocrit, the 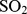 in arteries and veins, and vessel wall elasticity affect 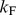 and 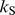 values. These physiological parameters are supposed to be stable in the the model; however, the validity is only assumed. In this study, the CWMD method was applied to the same experimental data, and the results agreed with those of the proposed method. Since the CWMD method is based on the optical multilayer model of the human head where the optical properties and layer structures of cephalic tissues are stable, the CWMD method itself is considered to be stable. Therefore, we assume that the proposed method is as stable as the CWMD method.

In pathological brains of ischemia [53] or altered rCBF response [54], the rCBF may increase less whereas the oxygen consumption in this tissue will increase in association with the neural activation. In these cases, the capillary blood will receive more deoxygenation and it will cause an increase or at least a smaller decrease of deoxyhemoglobin in a functional hemodynamic response. In fact, the increase in deoxyhemoglobin during activation in ischemic human brain was reported [53]. A disappearance of decrease in deoxyhemoglobin under the inhibition of a CBF response was observed in a rodent experiment [55]. Obviously we cannot use a universal 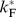 value in these pathological cases. Also, controversial studies on the functional hemodynamic response in young infants were reported [56]. There, many studies reported the increase in deoxyhemoglobins during neural activation. Such an increase in deoxyhemoblobin may be explained by a halting of the rCBF response in infants' immature brains. However, also unresolved is what 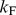 value will be suitable for infants, and how the 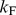 value changes over stages of development. In any cases, we may need to determine both 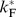 and 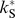 simultaneously, for example, by minimizing the mutual information. If further reliable detection is required, the CWMD or DOT method must be used. These can effectively detect hemodynamics in the cerebral layer even in these cases.

Some researchers observed nonlocalized hemodynamic changes similar to the functional component in terms of time range and modality [6]–[8]. Tissular and physiological origins of these hemodynamics are unknown. If any cephalic circulation change causes an increase in the capillary flow in the global cerebral cortex, it will cause a hemodynamic change similar to the functional hemodynamic modality, namely a prominent increase in oxyhemoglobin accompanied by a considerable decrease in deoxyhemoglobin. However, while a blood flow change in major vessels such as the middle cerebral artery was observed during the task execution [10], such a global capillary flow change has not been observed in the cerebral cortex. This type of global-task-evoked fluctuation is difficult to distinguish from a functional component in the proposed method; thus, it may degrade signal localization for the functional component. While viewing the functional component in Figure 6, responses were observed to some extent even when the ipsilateral finger was tapped. In most cases, however, these responses were somewhat smaller than the true functional signals that were observed when the contralateral finger was tapped. This suggests that such a circulation change does not seriously affect the detection of functional activation by the proposed method when a study subject is observed under mild physiological conditions. Further studies of this kind of cephalic circulation change are required to improve not only the proposed method but also other functional neuroimaging techniques such as fMRI.

A systematic method for fNIRS data analysis such as SPM for fMRI data analysis has not been established yet. For example, researchers have different understandings on the grand average of data obtained from different subjects. In this study, we consistently used individual data instead of their grand averages. Because individual data are differently scaled owing to the difference in the optical path length of subjects or optode positions [57], [58], we cannot calculate a precise average over subjects unless the optical path lengths are given. This is the primary reason why we did not indicate averages over subjects in Figures 4, 5, and 6. For the same reason, the t-tests in Table 2 were not corrected for multiple comparisons. In addition to the issue of scaling difference, we can observe noises that vary with subjects and optode positions in Figure 6. The differences in both scaling and noise violate the assumption of homoscedasticity, which is required in most correction techniques for multiple comparisons. In this case, only the comparison between left and right finger tappings can address this issue. Hence, we simply examined the difference in oxyhemoglobin change between the left and right finger tappings by the paired t-test, as shown in Table 2. However, we also noted that the significant level in the statistic corresponding to p = 0.05 could be increased to a certain degree if an overall multiple comparison is sufficiently conducted. Overcoming the issue relating to multiple comparisons mentioned here is crucial to establish a systematic data analysis method not only for group data but also for multichannel measurements in fNIRS.

Since fNIRS is a less invasive, easy-to-use technique compared to fMRI or PET, users expect multichannel fNIRS to be an alternative to such functional neuroimaging techniques. In usual CW fNIRS, a source-detector distance of 30 mm is used to detect the hemodynamic change in the cerebral gray matter layer. For this reason, commercially available multichannel CW fNIRS systems usually have an optode lattice configuration with a 30 mm pitch. However, in this case, the detectable cerebral hemodynamics area is limited to an area approximately half the size of the source-detector distance [4]. Therefore, the multichannel measurement with such an optodes configuration offers a spatially sparse sampling of hemodynamic changes. If we use it for the exploratory detection of an activation area, an intense but localized activation signal may be overlooked, but another area with a weaker signal may be falsely recognized as an activation area [59]. An improvement in the sampling density of multichannel fNIRS can be achieved by arranging duplicate optode lattices where one lattice is placed at the half-pitch shifted position of the other lattice [60]. Since the method proposed here is easily applicable to such a high density, multichannel system, we can simultaneously improve the spatial resolution and signal reliability.

## Conclusions

Based on the results and discussion above, we believe that the proposed method can separate the conventional fNIRS signal into functional and systemic components even when the task involves motion. By this simple method, users will be able to increase the reliability of fNIRS measurements without any modification in commercially available CW fNIRS instruments.
